# Effect of Robot-Assisted Therapy on Participation of People with Limited Upper Limb Functioning: A Systematic Review with GRADE Recommendations

**DOI:** 10.1155/2021/6649549

**Published:** 2021-07-31

**Authors:** Fernanda M. R. M. Ferreira, Maria Emília A. Chaves, Vinícius C. Oliveira, Jordana S. R. Martins, Claysson B. S. Vimieiro, Adriana M. V. N. Van Petten

**Affiliations:** ^1^Graduate Program in Mechanical Engineering, Universidade Federal de Minas Gerais, Bioengineering Laboratory, Av. Antônio Carlos, 6627, Pampulha, Belo Horizonte MG 31270-901, Brazil; ^2^Graduate Program in Physical Therapy, Centro Universitário UNA, Rua Aimorés, 1451, Lourdes, Belo Horizonte MG 30140-071, Brazil; ^3^Graduate Program in Rehabilitation and Functional Performance, Universidade Federal dos Vales do Jequitinhonha e Mucuri, Campus JK-MGT 367-Km 583, no. 5000, Alto da Jacuba, Diamantina MG 39100-000, Brazil; ^4^Graduate Program in Mechanical Engineering, Pontifícia Universidade Católica de Minas Gerais, Av. Dom José Gaspar, 500-Coração Eucarístico, Belo Horizonte MG 30535-901, Brazil; ^5^Department of Occupational Therapy, Universidade Federal de Minas Gerais, Av. Antônio Carlos, 6627, Pampulha, Belo Horizonte MG 31270-901, Brazil

## Abstract

**Background:**

Previous studies have suggested that robot-assisted therapy (RT) is effective in treating impairment and that it may also improve individuals' participation.

**Objective:**

To investigate the effect of RT on the participation of individuals with limited upper limb functioning (PROSPERO: CRD42019133880). *Data Sources*: PEDro, Embase, MEDLINE, CINAHL, Cochrane, AMED, and Compendex. *Inclusion Criteria*. We selected randomized or quasirandomized controlled studies comparing the effects of RT with minimal or other interventions on participation of individuals with limited upper limb functioning. *Data Extraction and Synthesis*. Methodological quality of the included studies was assessed using the 0-10 PEDro scale, and effect estimates were reported using standardized mean differences (SMDs) with 95% confidence intervals (CIs), and the certainty of the current evidence was assessed using the GRADE.

**Results:**

Twelve randomized controlled studies involving 845 participants were included. The estimates of medium effects between RT and minimal intervention (MI) at a short-term follow-up were pooled, but there are no short-term effects between RT and OI. Standardized differences in means were as follows: 0.6 (95% CI 0.1 to 1.2) and 0.2 (95% CI -0.0 to 0.4). There were also no effects of additional RT in the short- or medium-term follow-up periods. Standardized differences in means were as follows: -0.6 (95% CI -1.1 to -0.1) and 0.2 (95% CI -0.3 to 0.8). The methodological quality of the included studies potentially compromised the effect estimates of RT. The existing evidence was very low-quality with many confounding variables between studies.

**Conclusions:**

For patients with upper limb neurological dysfunction, low-quality evidence supports RT over MI in terms of improving individual participation in the short term. The existing low- to very low-quality evidence does not support RT over OI in either the short- or medium-term follow-up periods with respect to community participation.

## 1. Introduction

Robot-assisted therapy (RT) is an innovative approach to rehabilitation that involves intensive, repetitive, interactive, and individualized practice. RT includes a computerized control system and mechanical devices to promote motor learning and cortical reorganization required to enhance upper limb function [1, 2]. RT devices are reliable to measure kinematic and dynamic parameters during movements of the upper limb (e.g., movement quality, speed, time, direction, strength, and range of motion), allowing people's performance to be evaluated [3, 4].

RT has investigated individuals' poststroke (e.g., a common cause of movement disorders of the upper limbs) [5, 6], traumatic brain injury, spinal cord injury, and injuries to motor neurons, as well as certain neurological diseases, such as multiple sclerosis, cerebral palsy, Guillain-Barre syndrome, essential tremor, and Parkinson's disease [6–8]. The results from previous randomized controlled studies [7, 8] indicate that RT improves motor control (e.g., muscle activation patterns and movement) in the short and long term. For instance, in the short term, RT improved motor control by 3 points on a 0-36 Fugl-Meyer scale (shoulder-elbow subsection) compared with other interventions in patients with hemiparesis caused by upper motor neuron lesions [8]. The most recent systematic review with meta-analysis carried out by Mehrholz et al. [9] concluded that, based on high-quality evidence, robot-assisted arm training improved activities of daily living, arm function, and arm muscle strength. Moreover, previous systematic reviews of RT and individuals' poststroke reported improvement in neurophysiological aspects of the upper limb (mainly in the shoulder and elbow) [5, 10–15].

Previous studies suggested that RT was effective in treating impairments [5, 7, 8, 15, 16] and might also improve participation of the subjects [5]. Participation is a domain defined by the International Classification of Functioning, Disability and Health [17] that should be targeted during rehabilitation programs. Individuals' participation is defined as a function of improved performance during daily tasks and occupations. Some authors [17–19] advocated that RT can improve participation and activities of daily living, allowing individuals to perform training more independently. However, the effectiveness of RT on the participation of individuals with limited upper limb functioning is still unclear [7, 20]. The literature in this area has not previously been systematically collated [21]. A recent review suggests that studies were needed to investigate the effects of robotic therapy on participation [9]. Therefore, the aim of this review was to investigate the effects of RT on the participation of individuals with limited upper limb function. GRADE (Grading of Recommendations Assessment, Development and Evaluation) was used to summarize the strength of the existing evidence of the studies included.

## 2. Materials and Methods

The protocol of this review was registered at PROSPERO (CRD42019133880).

### 2.1. Search Strategy, Inclusion Criteria, and Selection of Studies

The search for relevant studies was conducted in PEDro (Physiotherapy Evidence Database), Embase (Excerpta Medica Database), MEDLINE (Medical Literature Analysis and Retrieval System Online), CINAHL (Cumulative Index to Nursing and Allied Health Literature), Cochrane (Cochrane Collaboration), AMED (Allied and Complementary Medicine Database), and Compendex (Compendex Engineering Index) without language, age, or date restrictions. In addition, the reference lists of previous systematic reviews in this field were hand searched. Searches were initiated on the 27^th^ of February 2020, and descriptors were related to “Robot-Assisted Therapy” (robotics, orthotic devices, bionic device, exoskeleton, robotic aided therapy, therapy computer-assisted, robot-assisted, robotics-assisted, self-help devices, robotic device, dynamic orthotic device, robot-mediated therapy, robot-supported, computer-assisted instruction, computer aided, computer-aided design, computer assisted, artificial limb, rehabilitation robotics, human-robot interaction, robot aided rehabilitation, robotic rehabilitation, orthosis, taping, splinting, assistive technology devices, and assistive device therapy); “upper limb” (upper extremity, arm, arm injuries, hand, hand injuries, shoulder, shoulder injuries, elbow, axilla elbow, forearm injuries, forearm, finger, finger injuries, wrist injuries, and wrist); and “Randomized controlled trials” (random allocation, double blind method, single blind method, placebo, random, controlled clinical trial, clinical trial, comparative study, evaluation study, follow-up study, prospective study, and crossover studies) (Appendix Supplementary 1).

After searches, duplicates were removed and relevant titles and abstracts were screened. Then, two independent reviewers (FMRMF and MEAC) assessed the potential full texts for our eligibility criteria. A third reviewer (AMVNV) resolved between-reviewer disagreements.

To be included, studies had to fulfil the following inclusion criteria: (1) be a randomized or quasirandomized controlled study; (2) investigate participants with limited upper limb function caused by any health condition regardless of age or gender who were inpatients or outpatients from any clinical/hospital care settings including primary, secondary, or tertiary services and community individuals; and (3) investigate the effect of the intervention of interest, which was RT when compared with minimal interventions (MIs) or other interventions (OIs) on individuals' participation. RT was defined as the application of any electronic, computerized control system connected to mechanical devices that were designed to perform human functions. We considered no interventions, sham, placebo, and waiting list as MIs. We considered any other active interventions other than RT, such as conventional therapy and physical therapy, as OIs. Studies investigating whether RT combined with OI enhances effects compared to OI stand-alone were also included. The outcome of interest in this review was individuals' participation, defined according to the International Classification of Functioning, Disability and Health recommendations [17]. We considered participation as the involvement of an individual in real-life situation [18]. Quality of life refers to individuals' feedback about their health condition or its consequence [18]. Quality of life was also included and pooled as part of participation to maintain consistency with previous studies that considered quality of life to be within the participation domain [22–24]. To assess the eligible outcomes, the reviewers followed the protocols reported by Sivan et al. [2].

### 2.2. Assessment of the Methodological Quality of the Included Studies

Two independent reviewers (FMRMF and MEAC) assessed the methodological quality of the included studies using the 0-10 PEDro scale [25] (PEDro score > 6), with higher scores indicating higher methodological quality. Disagreements were resolved by consensus. When available, we used the scores that were already on the PEDro database (https://www.pedro.org.au/) [26].

### 2.3. Data Extraction

The data extracted by two independent reviewers (FMRMF and MEAC) at baseline included the number of participants, mean age, sex (percentages of males and females), cause of the upper limb disorder and its duration, evaluated joints, type of RT, comparison groups, frequency, and total duration of treatment. The outcome data extracted included the sample size, mean, and standard deviation (SD) for each variable for each group at the short-, medium-, and long-term follow-ups, when available (Appendix Supplementary 2). The short-term effects were considered time points up to 3 months after the baseline. Measurements of the medium-term effects were considered time points over 3 months, but less than 12 months after the baseline. The long-term effects were considered time points of at least 12 months after the baseline. When multiple time points were available within the same follow-up period, the time point that was closest to the end of the intervention was considered [27].

If standard deviations (SDs) were not available in some included studies, the SDs were imputed from the 95% confidence interval (CI) [28, 29], standard error (SE) [28, 30, 31], and interquartile range [32, 33], and average values from other included studies were estimated with similar participants. In other included studies, data reported as the median and interquartile range [32, 33] were converted into the mean and standard deviation according to the method used by Wan et al. [34]. When a given study investigated two different RTs [31, 35] and OIs [36], we combined the groups following the previous reviews in this field [12, 13]. In one study [26], the outcome data for the medium-term effects were not available and were not included in the quantitative analysis.

When the studies assessed individuals' participation using more than one outcome measure [30, 32, 35], we chose the outcome measure that was most similar to those used in the other included studies. When studies provided outcome data in different domain scores of the SIS (Stroke Impact Scale) or SF-36 (Short Form-36 Health Survey), we used the data from the studies that were more consistent with our outcome of interest (participation), SIS, participation [37–40], mobility [31], and the physical health domain in the SF-36 [32].

### 2.4. Data Analysis

The random effects model was used to conduct meta-analysis for each specific health condition with estimates reported using standardized mean difference (SMD) with 95% confidence intervals (CIs). Homogeneity was assessed using the *I*^2^ statistic [41]. A study was considered to have low heterogeneity if *I*^2^ ≤ 50% and moderate to high heterogeneity if *I*^2^ > 50% [40]. Individual studies were also reported in forest plots when pooling was not possible. To determine the clinical relevance of RT, the effect sizes were assessed using Cohen's *d* thresholds: 0.2, 0.5, and 0.8 for small, medium, and large effects, respectively [41]. A funnel plot was used to investigate publication bias when at least 10 studies were pooled [27]. Meta-analyses were performed using Comprehensive Meta-Analysis, version 3.3.070.

The GRADE approach was used to summarize the overall quality of the evidence for each outcome [42]. Initially, the evidence was assumed to be high-quality, but the ratings were downgraded by one point if one of the following prespecified criteria was met: (1) low methodological quality (PEDro score < 6), (2) inconsistency of estimates among pooled studies (*I*^2^ > 50%) [27] or when the assessment was not possible (no pooling), (3) indirectness or poor description of the participants (over 50% of the studies did not describe the inclusion criteria), and (4) imprecision (pooling of <400 participants for each outcome) [27]. Two reviewers (FMRMF and MEAC) independently assessed the quality of the evidence, and a third reviewer (VCO) resolved any disagreements.

Subgroup qualitative analysis was conducted to investigate the impact of methodological quality issues on the pooled effects. Studies with PEDro scores of five or less out of ten were excluded. Metaregression was not possible because of the small number of included trials.

## 3. Results

**Figure 1:**
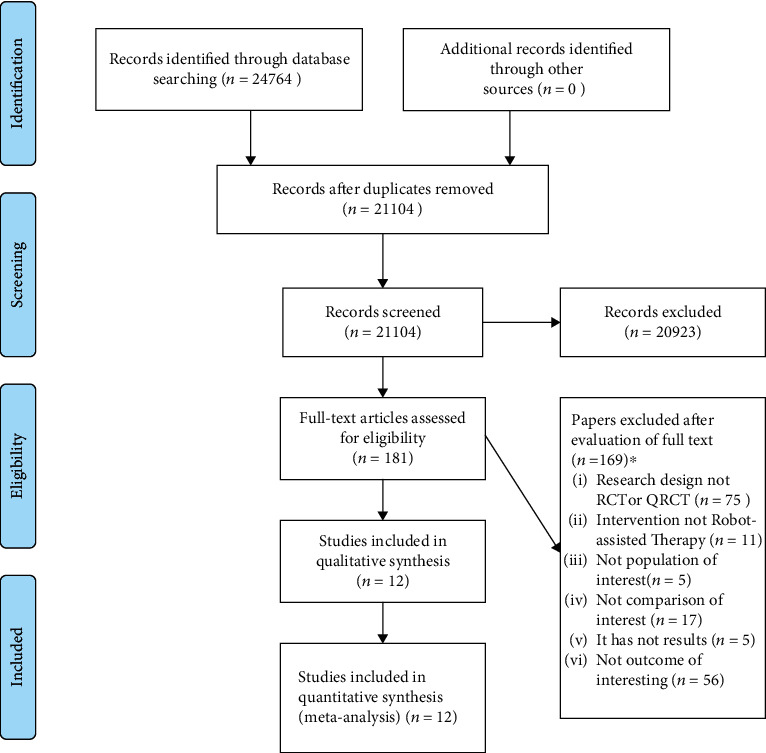
Flow diagram. ^∗^Papers may have been excluded for failing to meet more than one inclusion criterion. RCT = randomized controlled trial; QRCT = quasirandomized controlled trial.

After searches, titles and abstracts of 24,764 articles were screened, 181 potential full texts were assessed, and 12 original studies were included.  Figure 1 presents the flow diagram.

### 3.1. Characteristics of the Included Studies

All included studies were randomized controlled trials published in English between 2008 and 2019. The characteristics of the included studies are presented in Table 1.

Twelve included studies enrolled 845 participants of both genders. All studies reported neurological injury as the cause of limited upper limb functioning. The main cause of the neurological upper limb was stroke. Ten out of 12 studies included participants in the chronic phase poststroke [29–32, 35–38, 40], and one study [39] included participants in acute postinjury. Only one study [33] included participants with cerebral palsy. Six studies [28–31, 35, 36] compared RT with OI. Six studies [32, 33, 37–40] investigated the additional effect of RT with OI (the OI included physical therapy). One study compared RT with OI and compared RT with MI [28].

The duration of RT ranged from four [36] to 12 weeks [28, 40]. The frequency of intervention varied from two [33, 35] to five days [35] per week. The time spent per session of intervention ranged from 30 minutes [32] to 90-105 minutes [36]. On average, RT sessions were conducted three times per week over a total treatment duration of eight weeks. Eight different robotic devices were used: REAplan [33, 39], ARMin [29], UL-EXO7 [35], InMotion2 (commercial version of MIT-MANUS) [28, 30, 31], MIT-MANUS [40], Hand Mentor [37], Myomo e100 [38], Haptic Master [32], and Bi-Manu-Track [36].

Across all the studies, three different outcome measures were used to assess individuals' participation. Ten of 12 studies (83.33%) used the Stroke Impact Scale (SIS) [28–31, 35–40]. One study (8.33%) used Life Habits (Life H) [33], and another study (8.33%) used the Short Form-36 Health Survey (SF-36) [32]. All 12 included studies reported short-term effects. Two studies [32, 39] reported medium-term effects. No studies reported long-term effects.

### 3.2. Methodological Quality of Included Studies

The methodological quality of the included studies is presented in Appendix Supplementary 3. Based on the PEDro scale (0-10), the mean methodological quality score of the 12 studies was 6.5. Randomization, group similarity at baseline, group comparability, and reporting of precision/variability measures were presented in all included studies. The outcome measures for at least 85% of the participants were obtained in eight studies (66.66%). Intention-to-treat analysis, concealed allocation, and assessor blinding criteria were met in five (41.66%), six (50%), and eleven (91.66%) studies, respectively. The methodological quality issues were mainly related to participant and therapist blinding, and none of the studies met this criterion. None of the studies were double-blinded.

### 3.3. Effects of Robot-Assisted Therapy on Participation

#### 3.3.1. Robot-Assisted Therapy versus Minimal Intervention

**Figure 2:**
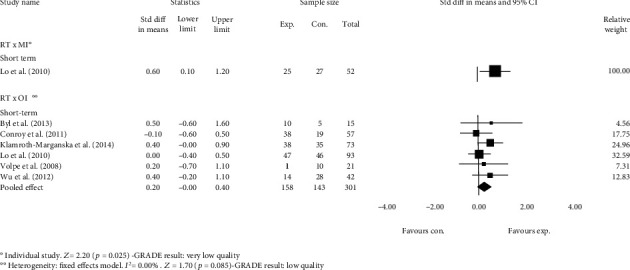
Standardized mean difference (95% CI) comparing the effects of RT alone versus MI and RT alone versus OI in the short term on the participation of individuals with limited upper limb functioning. RT = robot-assisted therapy; MI = minimal interventions; OI = other interventions; favours exp. = experimental group; favours con. = control group.

The estimates from one study [28] provided very low-quality evidence that RT has a medium effect on participation compared with MI (e.g., usual care, i.e., medical management and clinic visits as needed) in the short term. The SMD was 0.6 (95% CI 0.1 to 1.2, *p* = 0.025) (Figure 2). The evidence was downgraded from high quality to very low quality.

#### 3.3.2. Robot-Assisted Therapy versus Other Interventions

Pooled estimates from six studies showed low-quality evidence that RT has no short-term effect on participation compared with OI. The SMD was 0.2 (95% CI -0.0 to 0.4, *p* = 0.085) (Figure 2). The evidence was downgraded from high quality to low quality.

#### 3.3.3. Whether Robot-Assisted Therapy plus Other Intervention Enhances Effects of Other Intervention Alone

**Figure 3:**
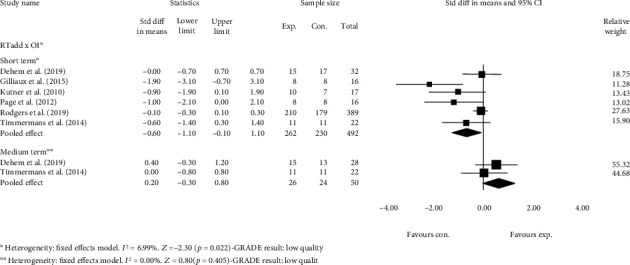
Standardized mean difference (95% CI) comparing additional effects of RT versus OI in the short and medium term on the participation of individuals with limited upper limb functioning. RT = robot-assisted therapy; OI = other interventions; favours exp. = experimental group; favours con. = control group.

Pooled estimates from six studies provided low-quality evidence that additional RT has a medium negative short-term effect on participation when compared with OIs alone. The SMD was -0.6 (95% CI -1.1 to -0.1, *p* = 0.022) (Figure 3). The evidence was downgraded from high to low quality.

Estimates from two studies [32, 39] provided low-quality evidence that additional RT has no effects on participation compared with OIs in the medium term. The SMD was 0.2 (95% CI -0.3 to 0.8, *p* = 0.405) (Figure 3). The evidence was downgraded from high quality to low quality. In all studies, the total therapy time in both groups was the same.

### 3.4. Subgroup Analyses

**Figure 4:**
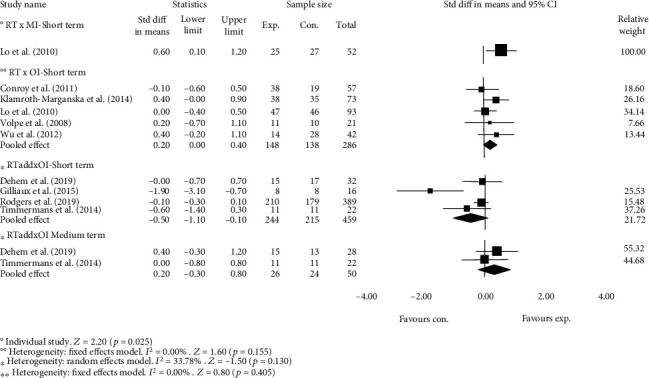
Subgroup analysis investigating the impact of methodological quality on estimated effects of RT at short- and medium-term follow-up. Favours exp. = experimental group; favours con. = control group.

Removing poor-quality studies (<6 out of 10 on the PEDro scale) from subgroup analysis suggested no impact on the reported estimates (Figure 4).

## 4. Discussion

RT has been frequently used to treat individuals with limited upper limb functioning because it allows performance and progress to be assessed with high reliability and accuracy [11, 43]. However, previous studies have focused on effects of RT on body structure and function [1, 2, 10, 15, 20] or on activity performance [5, 44]. These previous studies [1, 2, 10, 15, 20] have suggested clinically important effects of RT on structural and functional outcomes. This current study is the first systematic review of randomized controlled studies investigating the effects of RT on the participation of individuals with limited upper limb function.

In the short term, for patients with upper limb impairments, RT and additional RT did not improve participation compared with other interventions (OI). In the medium term, additional RT did not improve participation. In the short term, RT did significantly improve participation, showing medium clinical effect, compared to minimal interventions (MI). Unfortunately, this finding was based on only one study with a small sample.

The absence of short-term effects of RT as well as the absence of short- and medium-term effects of additional RT may be due to a few study variables. For example, different types of therapy were performed when using the robots. In some studies, repetitive task practice was included while other studies included intensive intervention strategies. Still others included conventional physical or occupational therapy. This variability in the interventions may have affected the results.

Another explanation for the absence of effects of RT alone and additional RT may be that most studies included patients with a chronic episode of the condition limiting upper limb function (e.g., at least 6 months poststroke). Research studies provide evidence that most spontaneous periods of central neural recovery occur during the first 3-6 months postinjury (e.g., poststroke). Additional measurable improvement in the chronic phase of recovery only occurs with patient motivation and commitment as well as continued opportunities to participate in rehabilitation [45].

Another possible explanation for the lack of significant differences in participation with RT alone or additional RT may have been due to the complexity of the outcome measures for participation. Participation is defined as an individual's involvement in a real-life situation [17]. It is a multidimensional component influenced by the interaction between the individual's capacity, their compliance with task practice, their self-motivation to practice comprehensive daily activities [24], or their actual previous experiences in a real context prior to the upper limb injury [18]. Further, in this real context, environmental factors (physical, social, and beliefs) may also influence the effects of RT [17].

Another limiting factor may have been that RT was performed in a laboratory environment. Laboratory practice may not sufficiently mimic an individual's real environment. Training in the laboratory environment may not allow generalization or learning transfer to other real-life environments. Kehayia et al. [46] suggested that interventions, even in the laboratory, must involve different contexts. These investigators [46] proposed that rehabilitation should take place in enabling physical and social environments in order to optimize social inclusion and the participation of individuals with physical disabilities.

Functional capacity, personal factors, family support, and physical or social environments can all play an important role in the *participation* of individuals with physical limitations [47]. Health care providers are reconsidering their approaches to rehabilitation to better meet patients' needs. Therapies involving new methods of addressing patients' functional skills in natural environments are being developed. Desjardins et al. [48] identified potential environmental factors (physical and social) that limit individuals' participation in real contexts (e.g., shopping centers, convenience stores, grocery stores, supermarkets, and shopping malls). Regardless of the rehabilitation strategy of interest, identifying environmental factors may help researchers develop technologies that overcome these limitations in real contexts and consequently improve individuals' participation.

Some clinicians may argue that RT is not appropriate because there are cheaper rehabilitative interventions. While RT may be expensive, it is too early to exclude this intervention from rehabilitation programs targeting participation outcomes. Previously, high-quality studies have shown clinically important effects of RT on body structure and function [15, 28–30, 32, 35, 36]. High-quality randomized controlled studies with large sample sizes are needed to determine more accurate estimates of the effects of RT on participation. The study populations need to be diverse, with statistical controls for population diversity and environmental factors. The studies also need to address how to make laboratory interventions better simulate translation into the community and society [21]. In addition, the effects of different RT devices need to be studied.

This review has some strength that it supports as recommendations. The evaluation of the methodological quality of the included studies was performed using the Physiotherapy Evidence Database (PEDro). The PEDro scale is widely used in systematic reviews in the rehabilitation area. In addition, two reviewers assessed study risk of bias independently. The statistical methods used in the data analysis were described in detail. For each result, the 95% confidence intervals and the *p* value for the magnitude of the effect were calculated. Because of the amounts of data available, heterogeneity was also assessed for each result. The potential limitations of this study include the inconsistency in the RT devices used across the included studies. The impact of the dose and duration of RT administered to the experimental and control groups was not controlled in this review. Due to small numbers of studies and small sample size, it was not possible to investigate the impact of the different robotic devices on participation. Another limitation was that the studies used different guidelines to interpret the SIS scores.

## 5. Conclusions

For individuals with chronic, limited upper limb function, in the short term, this systematic review provides low-quality evidence that RT improves individuals' participation more than minimal interventions (MI). This review provides no evidence that RT improves participation compared to other interventions (OIs) in either the short or medium term. The findings from this systematic review cannot be generalized to participants with acute upper limb neurological impairments.

## Figures and Tables

**Table 1:** Characteristics of the included studies (*n* = 12).

Study	Health condition	Source	Participants	Intervention	Duration and frequency	Outcome measures	Robotic device
Byl et al. [35]	Individuals with stroke, aged between 25 and 75 years, for at least 6 months	Not informed Location: California, San Francisco	*n* = 15 Exp RT = robotic therapy unilateral (*n* = 5; age 54.2 (SD 20.5); gender 1 F/4 M) Exp RT = robotic therapy bilateral (*n* = 5; age 65.2 (SD 5.4); gender 0 F/5 M) OI = task-specific repetitive training with a physical therapist (*n* = 5; age 59.3 (SD 6.8); gender 1 F/5 M)	Exp RT = robotic therapy unilateral Exp RT = robotic therapy bilateral OI = task-specific repetitive training with a physical therapist	Exp RT =90 min/session; 2/wk × 6 wk Exp RT =90 min/session; 2/wk × 6 wk OI =90 min/session; 2/wk × 6 wk	Quality of life = Stroke Impact Scale total range 0-100 Follow-up = postintervention (short term)	UL-EXO7 Bilateral and unilateral Shoulder, elbow, and wrist
Conroy et al. [31]	Adults with a diagnosis of chronic stroke, aged ≥18 years	Community-dwelling adults Location: not informed	*n* = 62 Exp RT = robotic therapy planar (*n* = 20; age 57 (SD 12; gender 9 F/11 M) Exp RT = robotic therapy combined planar with vertical (*n* = 18; age 60 (SD 13); gender 8 F/10 M) OI = intensive conventional arm exercise program (*n* = 19; age 56 (SD 6.3); gender 9 F/10 M)	Exp RT = robotic therapy planar Exp RT = robotic therapy combined planar with vertical OI = intensive conventional arm exercise program	Exp RT =60 min/session; 3/wk × 6 wk Exp RT =30 min planar robot+30 min vertical robot/session; 3/wk × 6 wk OI =60 min/session; 3/wk × 6 wk	Participation = Stroke Impact Scale (version 3.0) subscore mobility range 0-100 Follow-up = postintervention and 12 weeks later (short term)	MIT-MANUS Unilateral Shoulder and elbow
Dehem et al. [39]	Individuals with acute stroke < 1 month delay since stroke	Three Belgian inpatient rehabilitation centers: Cliniques universitaires Saint-Luc (Brussels), Centre Hospitalier Valida (Brussels), and Centre Hospitalier Neurologique William Lennox (Ottignies) Location: Belgium	*n* = 45 Exp RT add = robotic therapy+conventional therapy (*n* = 23; age 67.3 (SD 11.1); gender 12 F/11 M) OI = conventional therapy (*n* = 22; age 68.6 (SD 19.1); gender 12 F/10 M)	Exp RT add = robotic therapy+conventional therapy OI = conventional therapy	Exp RT add =25% robotic therapy 45 min/session+75% conventional therapy; 9 wk OI = 9 wk	Participation = Stroke Impact Scale subscore social participation range 0-100 Follow-up = postintervention and 24 weeks poststroke (medium term)	REAplan robot Unilateral Planar end effector
Gilliaux et al. [33]	Patients with cerebral palsy, a maximum age of 18 years	Recruited from a school for children with physical disabilities (Institut Royal de l'Accueil du Handicap Moteur, Brussels, Belgium) Location: Belgium	*n* = 16 Exp RT add = robotic therapy+conventional therapy (*n* = 8; age 10.8 (SD 4.6); gender N/A) OI = conventional therapy (*n* = 8; age (SD); gender N/A)	Exp RT add = robotic therapy+conventional therapy OI = conventional therapy	Exp RT = robotic therapy 2/wk+conventional therapy 45 min/session; 3/wk × 8 wk OI = 5/wk × 8 wk; 45 min/session	Participation = Life Habits range 0-10 Follow-up = postintervention (short term)	REAplan Unilateral Shoulder and elbow
Klamroth-Marganska et al. [29]	Patients with chronic stroke for at least 6 months, aged ≥18 years	Four clinical centers in Switzerland (Uniklinik Balgrist, Reha Rheinfelden, Zentrum für Ambulante Rehabilitation Zürich, and Zürcher Höhenklinik Wald) Location: Switzerland	*n* = 73 Exp RT = robotic therapy (*n* = 38; age 55 (SD 13); gender 17 F/21 M) OI = conventional therapy (*n* = 35; age 58 (SD 14); gender 10 F/25 M)	Exp RT = robotic therapy OI = conventional therapy	Exp RT =45 min/session; 3/wk × 8 wk OI =45 min/session; 3/wk × 8 wk	Quality of life = Stroke Impact Scale total (version 2.0) range 0-100 Follow-up = 8 and 34 weeks after the start of intervention (short term)	ARMin Unilateral Elbow wrist and hand
Kutner et al. [37]	Patients with subacute stroke, 3 to 9 months poststroke	Recruited from Emory University and the Cleveland Clinic Foundation Location: Cleveland, United States	*n* = 17 Exp RT add = robotic therapy+repetitive task practice (*n* = 10; age 61.9 (SD 13.4); gender 5 F/5 M) OI = repetitive task practice (*n* = 7; age 51 (SD 11.3); gender 2 F/5 M)	Exp RT add = robotic therapy+repetitive task practice OI = repetitive task practice	Exp RT add = robotic therapy 30 h+repetitive task practice 30 h; 3/wk OI =60 h; 3/wk	Participation = Stroke Impact Scale subscore social participation range 0-100 Follow-up = postintervention and 8 weeks later (short term)	Hand Mentor Unilateral Wrist and hand
Lo et al. [28]	Patients with stroke for at least 6 months, who were 18 years of age or older	Recruited veterans from four participating veterans affairs medical centers Location: United States	*n* = 127 Exp RT = robotic therapy (*n* = 49; age 66 (SD 11); gender 2 F/47 M) OI = intensive comparison therapy (*n* = 50; age 64 (SD 11); gender 2 F/48 M) MI = usual care (*n* = 28; age 63 (SD 12); gender 1 F/27 M)	Exp RT = robotic therapy OI = intensive comparison therapy MI = usual care (i.e., medical management and clinic visits as needed)	Exp RT =60 min/session, 3/wk × 12 wk OI =60 min/session 3/wk × 12 wk	Quality of life = Stroke Impact Scale total (version 3.0) range 0-100 Follow-up =12 and 36 weeks after randomization (short term)	MIT-MANUS Unilateral Shoulder, elbow, wrist, and hand
Page et al. [38]	Volunteers with stroke, for at least 12 months, aged between 21 and 75 years	Volunteers were recruited using approved advertisements distributed to local stroke support groups and outpatient rehabilitation clinics Location: not informed	*n* = 16 Exp RT add = robotic therapy+repetitive task practice (*n* = 8; age 59 (SD 12.9); gender 5 F/3 M) OI = repetitive task practice (*n* = 8; age 58.5 (SD 9.5); gender 0 F/8 M)	Exp RT add = robotic therapy+repetitive task practice OI = repetitive task practice	Exp RT add =60 min/session; 3/wk × 8 wk OI =60 min/session; 3/wk × 8 wk	Participation = Stroke Impact Scale subscore social participation range 0-100 Follow-up = 1 week postintervention (short term)	Myomo e100 Unilateral Elbow
Rodgers et al. [40]	Volunteers with stroke aged at least 18 years with moderate or severe upper limb functional limitation, between 1 week and 5 years after their first stroke	Participants were recruited from stroke units, outpatient clinics, day hospitals, community rehabilitation services, local stroke clubs, and primary care of four National Health Service (NHS) centers in the United Kingdom Location: United Kingdom	*n* = 770 Exp RT add = robotic therapy+usual care (*n* = 257; age 59.9 (SD 13.5); gender 101 F/156 M) OI = conventional therapy enhanced upper limb therapy+usual care (*n* = 259; age 59.4 (SD 14.3); gender 100 F/159 M) OI = usual care (*n* = 254; age 62.5 (SD 12.5); gender 101 F/153 M)	Exp RT add = robotic therapy+usual care OI = conventional therapy enhanced upper limb therapy (EULT) program based on repetitive functional task practice+usual care OI = usual care	Exp RT =45 min/session; 3/wk × 12 wk OI =45 min/session; 3/wk × 12 wk OI = received usual NHS care, which was provided by their local clinical service	Quality of life = Stroke Impact Scale total (version 3.0) range 0-100 Follow-up = postintervention and 12 weeks later (short term)	MIT-Manus robotic Gym (shoulder-elbow module, wrist module, and hand module integrated on to the shoulder-elbow module) Unilateral
Timmermans et al. [32]	Patients with chronic stroke for at least 8 months, aged between 18 and 85 years	Recruited from Adelante Rehabilitation Centre (Hoensbroek, NL) Location: Netherlands	*n* = 22 Exp RT add = robotic therapy+task-oriented training method (*n* = 11; age 61.8 (SD 6.8); gender 3 F/8 M) OI = arm-hand training program (*n* = 11; age 56.8 (SD 6.4); gender 3 F/8 M)	Exp RT add = robotic therapy+task-oriented training method OI = arm-hand training program	Exp RT add =30 min/session; 4/wk × 8 wk OI =30 min/session; 4/wk × 8 wk	Quality of life = SF-36 subscore physical health range 0-100 Follow-up = 8 and 24 weeks after the start of intervention (medium term)	Haptic Master Unilateral Shoulder and elbow
Volpe et al. [30]	Patients with stroke who had impaired arm and hand mobility for at least 6 months	Recruited at from outpatient clinic Location: not informed	*n* = 21 Exp RT = robotic therapy (*n* = 11; age 62 (SD 3); gender 3 F/8 M) OI = conventional therapy (*n* = 10; age 60 (SD 3); gender 3 F/7 M)	Exp RT = robotic therapy OI = conventional therapy	Exp RT =60 min/session; 3/wk × 6 wk OI =60 min/session; 3/wk × 6 wk	Quality of life = Stroke Impact Scale total (version 2.0) range 0-100 Follow-up = postintervention and 12 weeks later (short term)	MIT-MANUS Unilateral Shoulder and elbow
Wu et al. [36]	Individuals with unilateral chronic stroke, for at least 6 months	Not informed Location: not informed	*n* = 42 Exp RT = robotic therapy bilateral arm training (*n* = 14; age 55.13 (SD 12.72); gender 4 F/10 M) OI = therapist-based bilateral arm training (*n* = 14; age 57.04 (SD 8.78); gender 2 F/12 M) OI = conventional therapy (*n* = 14; age 51.30 (SD 6.23); gender 4 F/10 M)	Exp RT = robotic therapy bilateral arm training OI = therapist-based bilateral arm training OI = conventional therapy	Exp RT = 90-105 min/session; 5/wk × 4 wk OI = 90-105 min/session; 5/wk × 4 wk OI =90-105 min/session; 5/wk × 4 wk	Quality of life = Stroke Impact Scale total range 0-100 Follow-up = postintervention (short term)	Bi-Manu-Track Bilateral Forearm and wrist

*n* = sample size; SD = standard deviation; exp = experimental group; N/A = not available; M = masculine; F = feminine; RT = robot-assisted therapy; add = additional; OI = other intervention; wk = week(s); yr = year(s); min = minutes; h = hours.

## Data Availability

The data analysed during this systematic review are included in supplementary material files.

## Supplementary Materials

Supplementary 1 Appendix 1: full search strategy conducted on February 27^th^, 2020.

Supplementary 2 Appendix 2: extracted data for outcome measures.

Supplementary 3 Appendix 3: methodological quality of the included studies using the PEDro scale.

Supplementary 4 PRISMA checklist.
