# Supplementary material for: Effect of Robot-Assisted Therapy on Participation of People with Limited Upper Limb Functioning: A Systematic Review with GRADE Recommendations
Source: Occup Ther Int. 2021 Jul 31;2021:6649549. doi: 10.1155/2021/6649549 (PMC8349462; doi:10.1155/2021/6649549)
Supplement: Supplementary 1 — Appendix 1: full search strategy conducted on February 27th, 2020. [file 6649549.f1.docx]

**Appendix 1- Full Search strategy conducted on February 27^th^ 2020**

| ***OVID (Embase, Medline, Cochrane Central, Amed) Search Strategy***   1. Randomized Controlled Trials as Topic/ or randomized controlled trial$.mp. or Random Allocation/ 2. randomized controlled trial$.mp. or Randomized Controlled Trial/ 3. double blind method.mp. or Double-Blind Method/ 4. single blind method.mp. or Single-Blind Method/ 5. placebos.mp. or Placebos/ 6. ((singl$ or doubl$ or tripl$ or trebl$) adj25 (blind$ or mask$)).mp. 7. placebo$.mp. 8. random$.mp. 9. controlled clinical trial$.mp. or Controlled Clinical Trial/ 10. clinical trial$.mp. or Clinical Trial/ 11. comparative stud$.mp. 12. evaluation stud$.mp. 13. follow-up stud$.mp. or Follow-Up Studies/ 14. prospective stud$.mp. or Prospective Studies/ 15. crossover studies.mp. or Cross-Over Studies/ 16. Robotics/ or robot$.mp. 17. Orthotic Devices/ or orthotic$.mp. 18. orthos$.mp. 19. bionic device$.mp. 20. exoskeleton.mp. 21. robotic aided therapy.mp. 22. Therapy, Computer-Assisted/ or robot-assisted therapy.mp. 23. robot-assisted.mp. 24. robotics-assisted.mp. 25. Self-Help Devices/ or robotic device$.mp. 26. dynamic orthotic device$.mp. 27. robot-mediated therapy.mp. 28. robot-supported.mp. 29. Computer-Assisted Instruction/ or computer aided.mp. or Computer-Aided Design/ 30. computer assisted.mp. 31. artificial limb$.mp. or Artificial Limbs/ 32. rehabilitation robotics.mp. 33. human-robot interaction.mp. 34. robot-aided rehabilitation.mp. 35. robotic rehabilitation.mp. 36. orthesi$.mp. 37. upper limb$.mp. or Upper Extremity/ 38. upper extremities.mp. 39. arm$.mp. or Arm/ or Arm Injuries/ 40. hand$.mp. or Hand/ or Hand Injuries/ 41. shoulder$.mp. or Shoulder/ 42. Elbow/ or axilla elbow.mp. 43. Forearm Injuries/ or Forearm/ or forearm$.mp. 44. finger$.mp. or Fingers/ 45. Wrist Injuries/ or wrist$.mp. or Wrist/ 46. 1 or 2 or 3 or 4 or 5 or 6 or 7 or 8 or 9 or 10 or 11 or 12 or 13 or 14 or 15 47. 16 or 17 or 18 or 19 or 20 or 21 or 22 or 23 or 24 or 25 or 26 or 27 or 28 or 29 or 30 or 31 or 32 or 33 or 34 or 35 or 36 48. 37 or 38 or 39 or 40 or 41 or 42 or 43 or 44 or 45 49. 46 and 47 and 48   ***PEDro Search Strategy***   1. Abstract & Title: robot-assisted therapy OR robot OR exoeskeleton OR robot-supported OR orthosis 2. Therapy: orthosis, taping, splinting 3. Problem: not applicable 4. Body Part: not applicable 5. Subdiscipline: not applicable 6. Topic: not applicable 7. Method: Clinical trial 8. Author/Association: not applicable 9. Title Only: not applicable 10. Source: not applicable 11. Published Since: not applicable 12. New records added since: not applicable13.Score of at least: not applicable   ***CINAHL Search Strategy***   1. (MH "Clinical Trials+") OR "Randomized Controlled Trials as Topic/ or randomised controlled trial$.mp. or Random Allocation/" 2. (MH "Double-Blind Studies") OR "Double-Blind Method" 3. (MH "Single-Blind Studies") OR "Single-Blind Method" 4. placebo$ 5. (MH "Random Assignment") OR "random$" 6. clinical trial$ 7. (MH "Comparative Studies") OR "comparative studies" OR (MH "Prospective Studies+") 8. evaluation studies 9. Follow-Up Studies 10. Cross-Over Studies 11. (MH "Robotics") OR "robot$" 12. (MH "Orthoses+") OR "orthotic$" 13. "orthos$" 14. (MH "Assistive Technology Devices+") OR "bionic device$" 15. "exoskeleton" 16. (MH "Assistive Device Therapy (Saba CCC)") OR "robot-assisted therapy" 17. (MH "Assistive Device Therapy (Saba CCC)") OR "robot-assisted therapy" OR (MH "Therapy, Computer Assisted+") 18. "robot-assisted" 19. "robotics-assisted" 20. (MH "Assistive Technology Devices+") OR "Self-Help Devices" 21. "robotic device$" 22. "dynamic orthotic device$" 23. "robot-mediated therapy" 24. "robot-supported" 25. (MH "Computer Assisted Instruction") OR (MH "Computer Aided Design+") OR "Computer-Assisted Instruction" 26. (MH "Computer Aided Design+") OR "computer aided" 27. "computer assisted" 28. "artificial limb$" 29. "rehabilitation robotics" 30. "human-robot interaction" 31. "robot-aided rehabilitation" 32. "robotic rehabilitation" 33. "orthesis" 34. (MH "Upper Extremity+") OR "upper limb$" 35. (MH "Arm Injuries+") OR (MH "Arm") OR "arm$" 36. (MH "Hand Injuries+") OR "hand$" 37. (MH "Shoulder Injuries+") OR "shoulder$" 38. (MH "Elbow") OR "Elbow" OR (MH "Elbow Injuries+") 39. "axilla elbow" 40. (MH "Forearm") OR "Forearm" OR (MH "Forearm Injuries+") 41. (MH "Finger Injuries+") OR "finger$" 42. (MH "Wrist Injuries+") OR (MH "Wrist") OR "wrist$" 43. S1 OR S2 OR S3 OR S4 OR S5 OR S6 OR S7 OR S8 OR S9 OR S10 44. S11 OR S12 OR S13 OR S14 OR S15 OR S16 OR S17 OR S18 OR S19 OR S20 OR S21 OR S22 OR S23 OR S24 OR S25 OR S26 OR S27 OR S28 OR S29 OR S30 OR S31 OR S32 OR S33 45. S34 OR S35 OR S36 OR S37 OR S38 OR S39 OR S40 OR S41 OR S42 46. S43 AND S44 AND S45   ***Compendex Search Strategy***  ((Wrist*) OR (Finger*) OR (Forearm*) OR (axilla elbow) OR (Elbow*) OR (Shoulder*) OR (Hand*) OR (Arm*) OR (Upper Extremit*) OR (upper limb*)) AND ((Robotic*) OR (robot*) OR (Orthose*) OR (orthotic*) OR (Assistive Technology Device*) OR (exoskeleton) OR (Assistive Device Therapy) OR (robot-assisted therapy) OR (Therapy, Computer Assisted) OR (robot-assisted) OR (robotics-assisted) OR (Assistive Technology Device*) OR (Self-Help Device*) OR (robotic device*) OR (dynamic orthotic device*) OR (robot-mediated therapy) OR (robot-supported) OR (Computer Assisted Instruction) OR (Computer Aided Design) OR (computer assisted) OR (artificial limb*) OR (rehabilitation robotics) OR (human-robot interaction) OR (robot-aided rehabilitation) OR (robotic rehabilitation) OR (orthes*)) AND ((Randomized Controlled Trial*) OR (randomised controlled trial*) OR (Random*)) |
| --- |
