# Supplementary material for: Effect of Robot-Assisted Therapy on Participation of People with Limited Upper Limb Functioning: A Systematic Review with GRADE Recommendations
Source: Occup Ther Int. 2021 Jul 31;2021:6649549. doi: 10.1155/2021/6649549 (PMC8349462; doi:10.1155/2021/6649549)
Supplement: Supplementary 2 — Appendix 2: extracted data for outcome measures. [file 6649549.f2.docx]

**Appendix 2- Extracted data for outcome measures.**

Table 1. Extracted data for outcome measures

| **Study** | **Exercise** | | | **^*^Comparison** | | |
| --- | --- | --- | --- | --- | --- | --- |
|  | **Mean** | **SD** | **Sample** | **Mean** | **SD** | **Sample** |
| **(Exercise vs minimal intervention)** | | | | | | |
| ***Short-term*** |  |  |  |  |  |  |
| ^§^Lo et al. [28] | 4.61 | 11.80 | 25 | -3.03 | 12.16 | 27 |
| **(Exercise vs other intervention)** | | | | | | |
| ***Short- term*** | | | | | | |
| Byl et al. [35] | 136.95 | 31.94 | 10 | 122.80 | 26.69 | 5 |
| ^§^Conroy et al. [31] | 0.55 | 13.14 | 38 | 1.30 | 11.99 | 19 |
| ^‡^ Klamroth-Marganska et al. [29] | 1.42 | 3.29 | 38 | 0 | 3.29 | 35 |
| ^§^Lo et al. [28] | 6.31 | 11.52 | 47 | 5.77 | 11.33 | 46 |
| ^§^Volpe et al. [30] | 67.10 | 7.96 | 11 | 65.50 | 7.59 | 10 |
| Wu et al. [36] | 57.09 | 28.70 | 14 | 47.16 | 20.20 | 28 |
| **(Additional effects of Robot Assisted Therapy vs other intervention)** | | | | | | |
| ***Short-term*** |  |  |  |  |  |  |
| Dehem et al. [39] | 50 | 21.40 | 15 | 50.90 | 34.70 | 17 |
| þ Gilliaux et al. [33] | 113.50 | 24.68 | 8 | 154 | 17.32 | 8 |
| Kutner et al. [37] | 48.70 | 21.40 | 10 | 67.90 | 20.30 | 7 |
| Page et al. [38] | 31 | 6.98 | 8 | 37 | 4.38 | 8 |
| Rodgers et al. [40] | 47 | 25.90 | 210 | 49.20 | 23.80 | 179 |
| þ Timmermans et al. [32] | 58.40 | 12.69 | 11 | 71 | 27.58 | 11 |
| ***Medium- term*** |  |  |  |  |  |  |
| Dehem et al. [39] | 59.40 | 24.10 | 15 | 47.50 | 31.50 | 13 |
| þ Timmermans et al. [32] | 64 | 15.74 | 11 | 64 | 26.65 | 11 |

SD = standard deviation

**^*^**Comparison = other intervention

^‡^SD estimated based on confidence intervals

^§^SD estimated based on standard errors

þ SD estimated based on first and third quartile
