# Supplementary material for: Effect of Robot-Assisted Therapy on Participation of People with Limited Upper Limb Functioning: A Systematic Review with GRADE Recommendations
Source: Occup Ther Int. 2021 Jul 31;2021:6649549. doi: 10.1155/2021/6649549 (PMC8349462; doi:10.1155/2021/6649549)
Supplement: Supplementary 3 — Appendix 3: methodological quality of the included studies using the PEDro scale. [file 6649549.f3.docx]

**Appendix 3- Methodological quality of the included studies using PEDro scale.**

Table 3. Methodological quality of the included studies using PEDro scale

| Study | Random allocation | Concealed allocation | Groups similar at baseline | Participant blinding | Therapist blinding | Assessor blinding | <15% drop  outs | Intention-to-treat analysis | Between-group difference reported | Point estimate and variability reported | Total  (0 to 10) |
| --- | --- | --- | --- | --- | --- | --- | --- | --- | --- | --- | --- |
| Byl et al. [35] | Y | N | Y | N | N | Y | N | N | Y | Y | 5 |
| Conroy et al. [31] | Y | N | Y | N | N | Y | Y | N | Y | Y | 6 |
| Dehem et al. [39] | Y | Y | Y | N | N | Y | N | Y | Y | Y | 7 |
| Gilliaux et al. [33] | Y | N | Y | N | N | Y | Y | N | Y | Y | 6 |
| Klamroth-M. et al. [29] | Y | Y | Y | N | N | Y | Y | Y | Y | Y | 8 |
| Kutner et al. [37] | Y | Y | Y | N | N | N | N | N | Y | Y | 5 |
| Lo et al. [28] | Y | N | Y | N | N | Y | Y | Y | Y | Y | 7 |
| Page et al. [38] | Y | N | Y | N | N | Y | N | N | Y | Y | 5 |
| Rodgers et al.[40] | Y | Y | Y | N | N | Y | Y | Y | Y | Y | 8 |
| Timmermans et al. [32] | Y | Y | Y | N | N | Y | Y | Y | Y | Y | 8 |
| Volpe et al. [30] | Y | N | Y | N | N | Y | Y | N | Y | Y | 6 |
| Wu et al. [36] | Y | Y | Y | N | N | Y | Y | N | Y | Y | 7 |

Y = yes; N = no.
